# Both brain size and biological sex contribute to variation in white matter microstructure in middle‐aged healthy adults

**DOI:** 10.1002/hbm.26093

**Published:** 2022-10-03

**Authors:** Live Eikenes, Eelke Visser, Torgil Vangberg, Asta K. Håberg

**Affiliations:** ^1^ Department of Circulation and Medical Imaging Norwegian University of Science and Technology Trondheim Norway; ^2^ Nuffield Department of Clinical Neurosciences University of Oxford Oxford UK; ^3^ Donders Institute Radboud University Nijmegen Medical Centre Nijmegen The Netherlands; ^4^ Department of Clinical Medicine UiT The Arctic University of Norway Tromsø Norway; ^5^ PET Center University Hospital North Norway Tromsø Norway; ^6^ Department of Neuroscience Norwegian University of Science and Technology Trondheim Norway; ^7^ Department of Diagnostic Imaging, MR‐Center St. Olav's University Hospital Trondheim Norway

**Keywords:** AD, axons, brain size, CNS, gender, general population, ICV, MD, MRI, myelin, neuroimaging, older adults, RD, white matter organization

## Abstract

Whether head size and/or biological sex influence proxies of white matter (WM) microstructure such as fractional anisotropy (FA) and mean diffusivity (MD) remains controversial. Diffusion tensor imaging (DTI) indices are also associated with age, but there are large discrepancies in the spatial distribution and timeline of age‐related differences reported. The aim of this study was to evaluate the associations between intracranial volume (ICV), sex, and age and DTI indices from WM in a population‐based study of healthy individuals (*n* = 812) aged 50–66 in the Nord‐Trøndelag health survey. Semiautomated tractography and tract‐based spatial statistics (TBSS) analyses were performed on the entire sample and in an ICV‐matched sample of men and women. The tractography results showed a similar positive association between ICV and FA in all major WM tracts in men and women. Associations between ICV and MD, radial diffusivity and axial diffusivity were also found, but to a lesser extent than FA. The TBSS results showed that both men and women had areas of higher and lower FA when controlling for age, but after controlling for age and ICV only women had areas with higher FA. The ICV matched analysis also demonstrated that only women had areas of higher FA. Age was negatively associated with FA across the entire WM skeleton in the TBSS analysis, independent of both sex and ICV. Combined, these findings demonstrated that both ICV and sex contributed to variation in DTI indices and emphasized the importance of considering ICV as a covariate in DTI analysis.

## Graphical abstract

The results showed a similar positive association between intracranial volume (ICV) and fractional anisotropy (FA) in all major white matter tracts in men and women. Both men and women had areas of higher and lower FA when controlling for age, but after controlling for age and ICV, and when performing the analysis in the ICV‐matched sample, only women had areas with higher FA. Combined, these findings demonstrated that both ICV and sex contributed to variation in diffusion tensor imaging (DTI) indices and emphasized the importance of considering ICV as a covariate in DTI analysis.

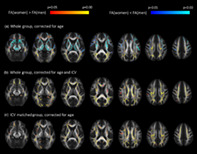

## 1 INTRODUCTION

Brain size is considered to scale with body size to preserve optimal processing characteristics while allowing control of a larger body. Sexual dimorphism in body size and brain size leads to larger brains in men than women on average. The degree to which characteristics of white matter (WM) as described by diffusion tensor imaging (DTI) indices are influenced by brain size only or if biological sex also plays a role remains an open question (Cox et al., 2016; David et al., 2018; Inano et al., 2011; Lebel et al., 2010; O'Dwyer et al., 2012; Pietrasik et al., 2020; Takao et al., 2011; Wu et al., 2011). We have previously reported similar WM volumes in men and women when matching for intracranial volume (ICV; Pintzka et al., 2015), suggesting that WM volume is mainly associated with brain volume, not sex. Still, WM microstructure investigated with DTI, could be influenced by ICV and/or sex, independent of ICV. As far as we know, this has not been examined before in ICV‐matched men and women.

A few previous studies have demonstrated that brain size is associated with proxies of WM microstructure (Takao et al., 2011; Takao et al., 2014; Warling et al., 2021). This association may influence the results of studies on sex differences in WM. Correcting for ICV significantly reduced the number of areas with sex differences (Takao et al., 2014) or lead to no significant sex‐differences in DTI indices (FA and MD) (Choi et al., 2010). Most studies, however, do not correct for ICV. Actually, the literature on sex differences in WM is conflicting, demonstrating higher FA in men, higher FA in women, a combination of both higher FA in men and women in different areas of the brain, or no differences between men and women (Cox et al., 2016; Inano et al., 2011; Lebel et al., 2010; O'Dwyer et al., 2012; Pietrasik et al., 2020; Wu et al., 2011). The results from the studies correcting for brain size suggests that differences in ICV might explain the sex differences in WM‐diffusion indices reported in the literature.

With increasing age, WM axons and myelin sheaths undergo degeneration as shown in postmortem human and animal studies (Aboitiz et al., 1996; Marner et al., 2003; Meier‐Ruge et al., 1992; Peters, 2002; Tang et al., 1997). In vivo DTI studies in humans show that DTI indices are correspondingly affected by age (Beck et al., 2021; de Groot et al., 2015; de Groot et al., 2016; Lebel et al., 2012; Molloy et al., 2021; Salami et al., 2012; Sexton et al., 2014; Sullivan et al., 2010). Although there is consensus in the literature that aging markedly reduces DTI derived proxies of WM microstructural integrity with a notable decrease in FA starting from around the age of 40–50 (Lebel et al., 2012), the anatomical localization of these changes varies between studies. Many studies report an anterior–posterior gradient of age‐related changes in DTI derived indices of WM microstructure (Bennett et al., 2010; Burzynska et al., 2010; Cox et al., 2016; Michielse et al., 2010; Salat et al., 2005) consistent with the last‐in‐first‐out hypothesis (Brickman et al., 2012). Other studies find a superior–inferior gradient of age‐related changes on DTI (Sexton et al., 2014; Sullivan et al., 2010; Zahr et al., 2009). A third model proposes selective deterioration of specific WM tracts particularly sensitive to the effect of aging based on DTI results (Greenwood, 2000; Salat, 2011). DTI studies have also shown that reductions in FA can serve as an early sign of neurodegenerative diseases such as Alzheimer's disease and small vessel disease (Finsterwalder et al., 2020; Luo et al., 2020), introducing additional complexity. Since the reductions in FA by normal aging and neurodegenerative diseases can appear at the same time and in the same regions of the brain, it is of great importance to elucidate the spatial patterns on DTI indices considered to reflect WM microstructural change related to healthy aging (Cox et al., 2016; Molloy et al., 2021). Most DTI studies investigating WM microstructure of the aging brain have been performed in samples with a wide age range incorporating the rise and decline in FA (3–6 decades and covering the age range of 5–100 years). So far, no study has performed a dedicated DTI investigation in healthy aging adults in the period coinciding with early aging, that is, between 50 and 66 years of age. Such a narrow age span allows for studying aging per se, without the contributions from the rapid changes in WM microstructure in early adulthood.

This study aimed to evaluate the associations and interactions between ICV, sex, and age on the WM microstructure from DTI in a large population‐based study of healthy aging individuals between the ages of 50 and 66 in the Nord‐Trøndelag health survey (HUNT) MRI study. Based on the preceding discussion, we predicted that increasing ICV was associated with higher FA and lower MD and RD values. Furthermore, we predicted that the differences in WM microstructure on DTI can be explained primarily by ICV, not sex. Likewise, we predicted that increasing ICV, rather than sex, was associated with larger tract volumes, similar to total cerebral WM volume (Kijonka et al., 2020; Pintzka et al., 2015). To separate the effects of sex and ICV on DTI indices associated with WM microstructure, we corrected for ICV, ICV and sex, or only sex in the entire sample. In addition, we stratified men and women into ICV‐matched groups to study sex differences in the DTI parameters without ICV as a confounding factor. We investigated DTI parameters derived from a voxel‐based method, that is, tract‐based spatial statistics (TBSS) providing a high certainty of performing the analysis in the center of common WM tracts and minimizing partial volume effects, and a semiautomated tractography region of interest (ROI) approach retrieving information from both the central and peripheral parts of the tracts. Methodological differences in DTI acquisition and analysis could possibly explain some of the discrepancies in the literature regarding sex and age‐related WM microstructural differences/associations (Håberg et al., 2015; Hollund et al., 2017; Lawrence et al., 2020). By including these two DTI analyses, we aimed to retrieve complementary information regarding proxies of WM microstructure to elucidate how differences in methodological approaches could explain discrepancies in the literature.

## 2 MATERIALS AND METHODS

### 2.1 Subjects

The participants were drawn from the population‐based HUNT stud, where all inhabitants ≥20 years in the county of Nord‐Trøndelag, Norway, were invited to participate in three surveys: HUNT 1 (1985–1987), HUNT 2 (1995–1997), and HUNT 3 (2006–2008) (Holmen et al., 1990; Holmen et al., 2003; Krokstad et al., 2013). Of 14,033 individuals aged 50–66, who had participated in HUNT 1, 2, and 3, and lived within 45 min of travel from the city of Levanger, 1560 were eligible to participate in the HUNT MRI study. Criteria for exclusion were limited to standard MRI contraindications and weight >150 kg. This left 1488 individuals that were invited to participate, of which 1087 gave informed consent. Of those, 81 did not undergo complete MRI examinations for various reasons (technical problems, no show, etc.). A total of 1006 successful MRI examinations were thus completed in 530 women (mean age 58.8 ± 4.3) and 476 men (mean age 59.2 ± 4.2). The HUNT‐MRI cohort (*n* = 1006) has been compared with those who were invited but did not participate (*n* = 554) and those who were eligible but not invited due to >45 min travel time from the city of Levanger (*n* = 12,473) (Honningsvag et al., 2012). The groups were not widely different, but the HUNT MRI cohort had a higher level of education, were less likely to be obese and have hypertension. Overall, the HUNT‐MRI participants are considered to constitute a representative sample of 50–66‐year‐old adults.

Of the 1006 MRI examinations, 97 had missing DTI (lack of or interrupted DTI acquisition) and 32 were excluded due to DTI artifacts (ghosting, signal voids, or excessive motion). Of the 877 good quality DTIs, another 65 individuals were excluded due to pathology (Håberg et al., 2016). In total, 812 DTI scans, 429 women (mean age 58.5 ± 4.3) and 383 men (mean age 59.2 ± 4.1), were suitable for further WM analysis. Since 20% of the HUNT‐MRI participants were excluded due to missing data, artifacts, and pathology, the 812 individuals included in this study were compared with the 194 individuals that were excluded regarding age, education, and sex. Age was slightly lower in those included (58.6 years vs. 59.6 years) (*p* = .039, independent *t* test), while education (*p* = .786, independent samples Mann–Whitney *U* test) and sex (*p* = .847, chi‐square test for homogeneity) were similar.

In this study, only age at time of MRI and sex were included in the analyses.

### 2.2 Image acquisition

All MRI examinations were acquired on the same 1.5 T GE scanner (Signa Hdx) equipped with an eight‐channel head coil using the same comprehensive scan protocol (Håberg et al., 2016). In the current study, the T1‐weighted volume, T2 weighted transversa, and DTI scans were used. The T1‐weighted volume was acquired with TR = 10.14 ms, TE = 4.04 ms, flip‐angle 10°, number of averages = 1, FOV 256 × 256 mm, acquisition matrix 256 × 256, slice thickness of 1.2 mm with no gap, giving a resolution of 1 × 1 × 1.2 mm^3^. The T2 weighted sequence was acquired with TR = 784 ms, TE = 95 ms, FOV = 230 mm, slice thickness = 4 mm, gap 1 mm, number of averages 2, number of slices 27, matrix 512 × 320, giving an inplane resolution of 0.45 × 0.45 mm^2^. The DTI sequence was a single‐shot balanced‐echo EPI sequence acquired in 40 noncollinear directions with *b* = 1000 s/mm^2^ and 5 *b* = 0 images using the following parameters: TR = 13,500 ms, TE = 104 ms, number of averages 1, FOV 240 × 240 mm, slice thickness 2.5 mm with no gap, acquisition matrix 96 × 96. The images were zero‐padded in *k*‐space to 256 × 256, giving a reconstructed resolution of 0.9275 × 0.9375 × 2.5 mm^3^. Sixty transversal slices with no gap were acquired giving full brain coverage.

### 2.3 DTI analysis

DTI analysis was performed with two methods; TBSS (FSL, The Oxford Centre for functional MRI of the Brain [FMRIB], Oxford, UK; www.fmrib.ox.ac.uk/fsl) and a semiautomated tractography method (Visser et al., 2011). Common to both methods: motion and eddy current distortions were minimized by registration of the DTI acquisition to the first *b* = 0 image using affine registration (“eddy_correct” in FSL).

#### 2.3.1 Tract‐based spatial statistics

The brain was extracted using Brain Extraction Tool (part of FSL). FMRIB's Diffusion Toolbox was used to fit a diffusion tensor model to the raw diffusion data in each voxel, and voxelwise maps of fractional anisotropy (FA), mean diffusivity (MD), axial diffusivity (AD), and radial diffusivity (RD) were calculated. Voxelwise statistical analysis of the diffusion data was performed using TBSS (Smith et al., 2006). Briefly, all subjects' FA data were aligned to the 1 × 1 × 1 mm^3^ FMRIB58_FA standard space atlas using a nonlinear registration. A mean FA image was created from all the aligned FA images and thinned to create a skeletonized mean FA representing the centers of all tracts common to all the subjects in the analysis. The mean FA skeleton was thresholded to FA ≥0.2 to include the major WM pathways, but excluded peripheral tracts and cortical gray matter. Each subject's aligned FA data were then projected onto the skeleton by searching perpendicular from the skeleton for maximum FA values in each individual's FA maps. Statistical comparisons of the FA, MD, AD, and RD maps are then restricted to voxels in the WM skeleton.

In all the TBSS statistics presented, the background image is the mean FA map of all subjects in the study. The images are shown in radiological convention, that is, the subjects' right side is on the left side of the images.

#### 2.3.2 Semiautomated tractography segmentation method

##### Tractography

The Camino package (Cook et al., 2006) was used for diffusion analysis and generation of streamlines for the semiautomated tractography segmentation method. Q‐ball reconstruction (Tuch, 2004) was used to parameterize the voxel diffusion profiles. Spherical harmonics up to fourth order were used as basis functions. Up to three principal diffusion directions were determined in each voxel and these were used as a basis for tractography. Streamlines were generated using the interpolated deterministic streamlining method, as implemented in Camino, with an FA threshold of 0.20. All voxels with an FA value greater than 0.25 were used as seed voxels.

##### Nonlinear registration

The mean *b* = 0 volumes for all subjects were affine registered to the MNI152 template. A custom group template was created by averaging the registered volumes. The original *b* = 0 volumes were then nonlinearly registered to the group template. FLIRT and FNIRT (FSL) were used in these registration steps. Using the deformation fields produced by FNIRT, the streamlines were warped from subject space to the group template.

##### Clustering

A clustering approach previously described in Visser et al. (2011) was used to find consistent bundles of streamlines across subjects, clustering the streamlines based on their pairwise distances. Before clustering, all streamlines were linearly resampled to 25 points, and the streamlines from all 812 subjects were concatenated. Clustering was performed on the merged data set consisting of streamlines from all subjects, allowing for the identification of consistent clusters across all subjects. The multisubject data set was randomly partitioned into subsets of 10,000 streamlines to allow for processing the complete set of data. In each of these subsets, 250 clusters were identified using hierarchical clustering (Johnson, 1967; Ward, 1963). The hierarchical clustering process is based on repeatedly finding clusters with the lowest mutual distance and merging them until 250 clusters are identified. The clustered subsets were then combined to obtain segmentations with the same number of clusters for the full data set, using a distance‐based matching procedure to find corresponding labels across subsets. The clustering step was repeated 100 times with different random partitions to obtain a stable segmentation by selecting the cluster assignments that occurred most often for each streamline and to find statistics indicating the consistency of these assignments between repetitions. WM structures were then identified in 10 randomly selected subjects by manually extracting sets of clusters to form the 8 following tracts: corpus callosum (CC), cingulum (CG), superior longitudinal fasciculus (SLF), inferior longitudinal fasciculus (ILF), inferior fronto‐occipital fasciculus (IFO), corticospinal tract (CST), optical radiation (OR), and uncinate fasciculus (UNC). These clusters were consistent across the 812 individuals, and the corresponding clusters were extracted with pruning (thresholding) and concatenated to form the 8 fiber tracts for all 812 subjects. After the segmentation, all the tracts were visually inspected, and some were excluded because they did not pass the visual quality control. Excluded tracts were 11 CG, 23 CC, 1 CST, 4 SLF, and 30 OR. The different tracts were excluded case wise, leaving a different number of individuals in each of the eight tracts. ROIs were made for the extracted fiber tracts and converted into each subject's DTI space to extract mean FA. WM tract volume was calculated for each WM fiber tract by summing the number of voxels containing at least one streamline and multiplying by voxel volume (measured in 10^4^ × mm^3^). It is important to note that this value reflects the number of voxels within the tract that exceeded the tracking FA threshold of 0.2 and might deviate from the actual volume of the WM pathway (Lebel et al., 2012).

#### 2.3.3 DTI head motion

Some studies have reported differences in head motion between men and women during MRI scanning (Huijbers et al., 2017; Van Dijk et al., 2012), which may lead to spurious group differences in the DTI indices. We, therefore, checked whether the average head motion during the DTI acquisition differed significantly between men and women by computing the scan‐to‐scan root mean squared motion (rms) (Jenkinson, 1999) from the affine transformation matrices made by “eddy_correct” using the “rmsdiff” program in FSL. The motion parameters were averaged over the diffusion‐weighted volumes (rms_mean_) for each individual. Differences in motion between men and women were tested using a two‐sided *t* test.

### 2.4 Intracranial volume

ICV (measured in ml) was estimated from the T1 and T2 weighted MR images in SPM8 (rel. 5236) (http://www.fil.ion.ucl.ac.uk/spm) using an upgraded automated version of the reverse brain mask method (Hansen et al., 2015). The role of ICV on the DTI parameters was investigated across all included HUNT‐MRI participants and in an ICV‐matched subgroup in order to study sex differences in the DTI parameters without ICV as a confounding factor. The ICV‐matched subgroup was formed by selecting pairs of men and women with an ICV that differed less than 10 ml. A total of 144 pairs of ICV‐matched men (mean age 51.7 ± 4.5, range 51.7–66.8) and women (mean age 58.2 ± 4.2, range 50.5–66.3) were found.

### 2.5 Statistical analyses

#### 2.5.1 Tract‐based spatial statistics

Randomise (part of the FSL program package) was used to perform voxelwise statistics on the skeletonized DTI data from TBSS. Multiple linear regression models, one for each DTI parameter (FA, MD, AD, and RD) as the dependent variable, and age, sex, and ICV as independent variables were used to study the relationship between the DTI parameters and ICV (corrected for sex and age), sex (corrected for age, and for age and ICV), and age (corrected for sex, and for sex and ICV). Interaction terms between ICV, sex, and age (i.e., ICV × sex, ICV × age, and sex × ICV × age) were initially added to the models, but since no interactions were found in any of the analysis, the interaction terms were removed. Randomise was used for permutation‐based testing (5000 permutations) and statistical inference (Winkler et al., 2014) with correction for multiple comparisons performed with threshold‐free cluster enhancement (TFCE) (Nichols & Holmes, 2002). Covariates were mean centered in the analysis. Significance levels for t tests were set at *p* < .05. Cohen's *d* (*d*) effect size was calculated voxelwise on the skeleton for the group differences between men and women in the whole sample and in the ICV‐matched men and women analysis. Mean Cohen's *d* was calculated separately for the statistically significant voxels from the FA, MD, AD, and RD analysis and reported in the results, while a voxelwise map of Cohen's *d* can be found in the supplementary materials for the FA, MD, AD, and RD analysis. We considered 0.2 a small effect, 0.5 a medium effect, and 0.8 a large effect. Unstandardized beta estimates were calculated voxelwise on the skeleton for the ICV‐DTI and age‐DTI associations by using the fsl_glm command in FSL. Mean beta estimates were calculated separately for the statistically significant voxels from the FA, MD, AD, and RD analysis and reported in the results, while a voxelwise map of the beta estimates can be found in the supplementary materials for the FA, MD, AD, and RD analysis.

#### 2.5.2 Semiautomated tractography ROIs

Statistical analyses for the ICV and tractography ROIs were performed in SPSS 21.0 (SPSS Inc., Chicago, IL). ICV group differences in men and women were analyzed with Student's *t* test, while linear regression was used to check whether ICV was associated with age in men and women separately. In the WM pathways that were tracked separately for the left and right hemispheres (SLF, ILF, IFO, CST, OR, and UNC), linear mixed models were used to study interaction effects between sex, hemisphere, and age on FA and volume. To reduce the total number of analyses in the study, FA was the only DTI‐index extracted from the tractography ROIs since this is the most commonly used DTI index. Since FA in the left/right hemispheres were found to interact with both sex and age, the tractography results for SLF, ILF, IFO, CST, OR, and UNC were analyzed separately for each hemisphere.

For the tractography (ROI) results for CC, CING and left and right SLF, ILF, IFO, CST, OR, and UNC, the same multiple linear regression models as for the TBSS analyses (univariate general linear model) were used to study the effects of ICV, sex, and age, including interaction effects, on mean FA and volume in the whole group. No interaction was present between ICV, sex, and/or age, and women and men were combined in the analysis between ICV and sex and between age and sex. The significance threshold was set at *p* < .05, corrected for multiple comparisons using the Benjamini–Hochberg method (Benjamini & Hochberg, 1995). The variables were grouped into three “families” that were treated separately (ICV effects, sex effects, and age effects), and tests within each family were corrected for multiple comparisons. The effect size was calculated as partial eta squared, where 0.01 constitutes a small effect, 0.06 a medium effect, and 0.14 a large effect.

### 2.6 Ethics

The study protocol was approved by the Regional Committee for Medical Research Ethics, and written informed consent was obtained from the participants.

## 3 RESULTS

### 3.1 Head motion

The average head motion during DTI acquisition (rsm_mean_) was 0.263 ± 0.055 mm for men and 0.256 ± 0.062 mm for women and was not significantly different (*p* = .098, *t* = −1.655). Head motion was therefore not included as a covariate in the TBSS or tractography ROI analysis.

### 3.2 DTI indices and ICV

#### 3.2.1 Intracranial volume

**FIGURE 1:**
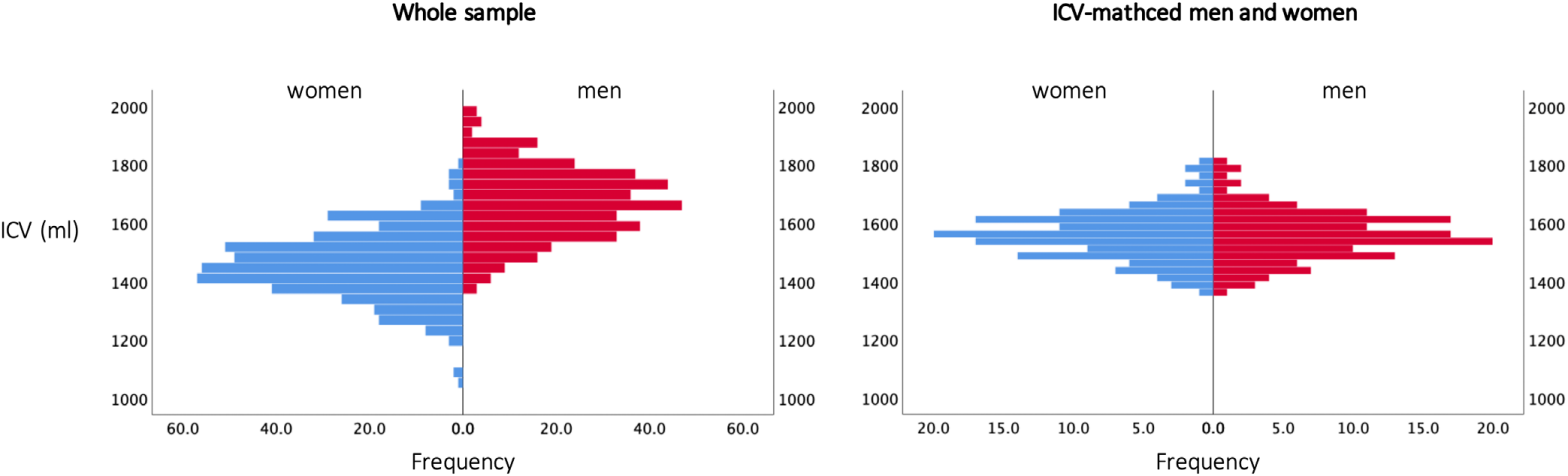
Histograms of intracranial volumes (ICVs) in the whole sample (left) and in the ICV‐matched (right) women (blue) and men (red)

**FIGURE 2:**
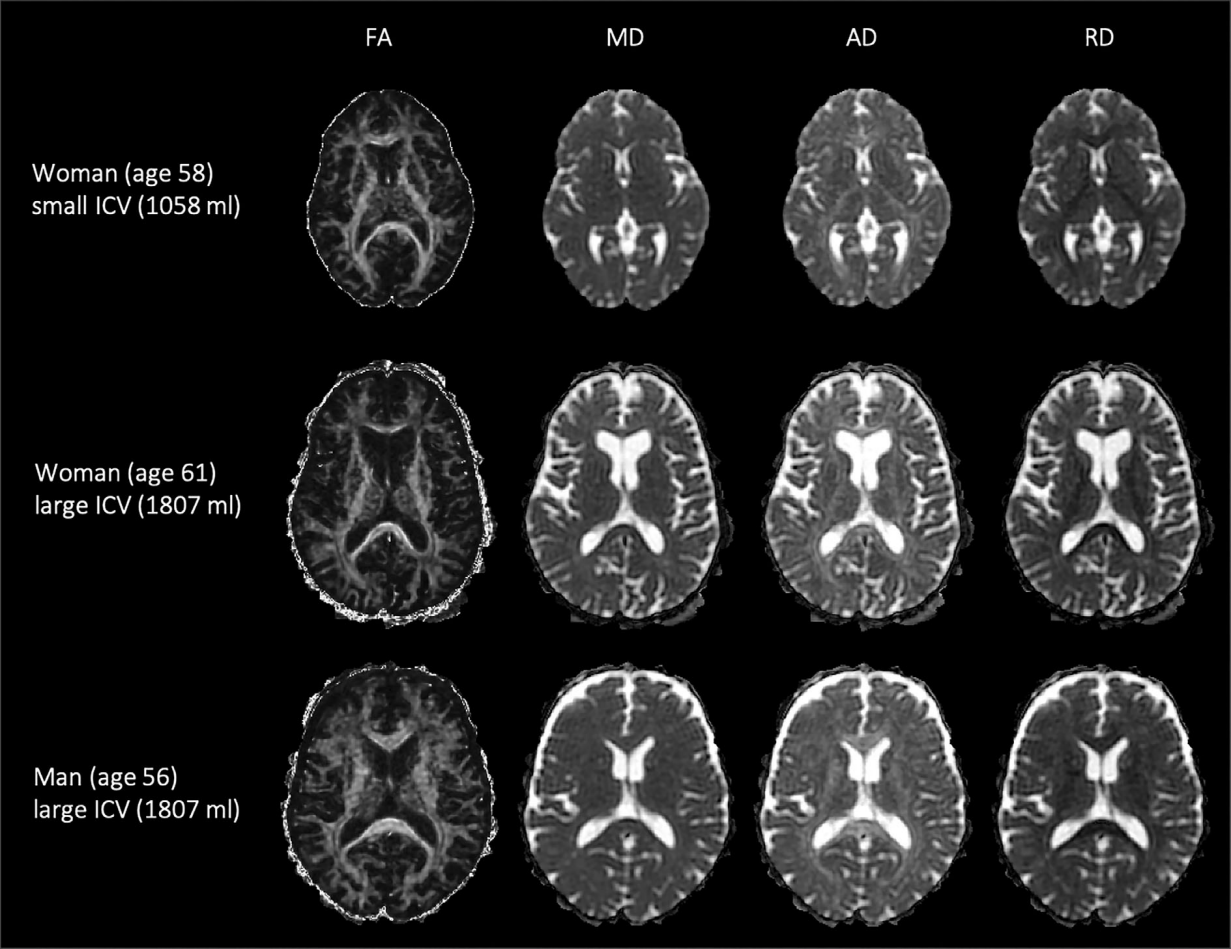
Fractional anisotropy (FA), mean diffusivity (MD), axial diffusivity (AD), and radial diffusivity (RD) images of three of the individuals in the study. The examples show the woman with the smallest intracranial volume (ICV; top row), the woman with the largest ICV (middle row), and a man with a similar ICV as the woman with the largest ICV (bottom row).

ICV was significantly larger in men (1667.9 ± 120.2 ml) compared to women (1455.9 ± 115.8 ml) in the whole group (*p* < .001, *t* = 25.6), while ICV was similar for men and women in the ICV‐matched groups (women: 1560.5 ± 85.3 ml, and men: 1560.4 ± 85.4 ml, *p* = .995, *t* = 0.017) (Figure 1). ICV did not change with age in men (*p* = .602) or in women (*p* = .402). FA, MD AD, and RD images of the woman with the smallest ICV, the woman with the largest ICV, and a man with a similar ICV as the woman with the largest ICV is demonstrated in Figure 2.

#### 3.2.2 Tract‐based spatial statistics

**FIGURE 3:**
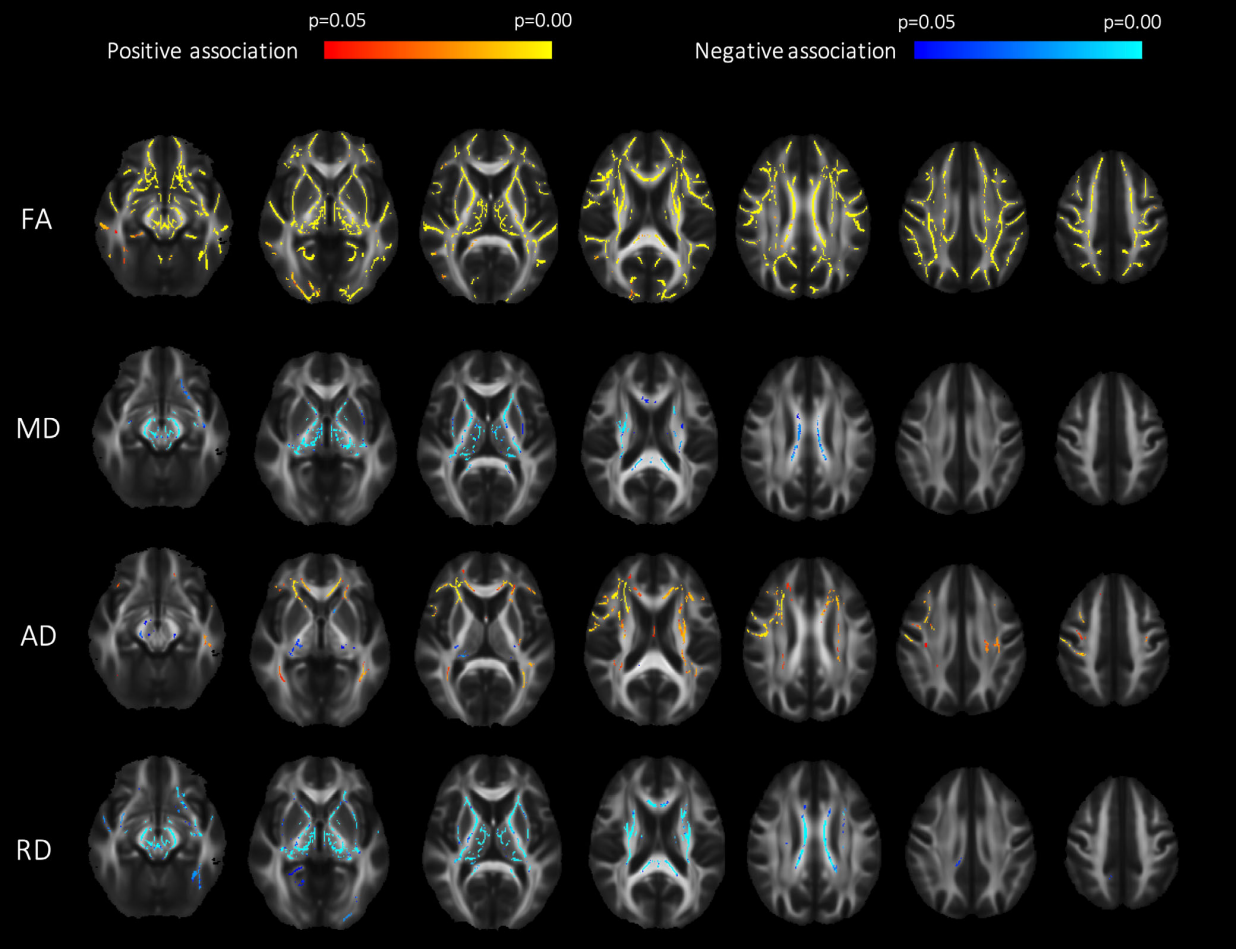
Effect of intracranial volume (ICV) on white matter microstructure. A positive association (red/yellow) was found between fractional anisotropy (FA) and ICV in all major white matter tracts in women and men jointly, while a negative association was found between ICV and mean diffusivity (MD). For axial diffusivity (AD), both positive (red/yellow) and negative (blue/light blue) associations were found. For radial diffusivity (RD), negative associations were uncovered in deep central parts of the brain (blue/light blue). All analyses were corrected for age, sex, and multiple comparisons, and statistical significance level set to *p* < .05.

The TBSS analysis demonstrated a positive association between ICV and FA for women and men in all major WM tracts (65% of all voxels in the skeleton) (*p* < .05, corrected for age, sex, and multiple comparisons, *β* = 4.7 × 10^−8^) (Figure 3). A negative association was found between ICV and MD in 48% (*β* = −6.2 × 10^−11^) and between ICV and RD in 76% (*β* = −6.1 × 10^−11^) of voxels with a positive association between ICV and FA. For AD, both positive (*β* = 9.2 × 10^−11^) and negative associations (*β* = −1.2 × 10^−10^) were found in 43 and 15% of the skeleton voxels with positive association between ICV and FA, respectively.

#### 3.2.3 Tractography

**FIGURE 4:**
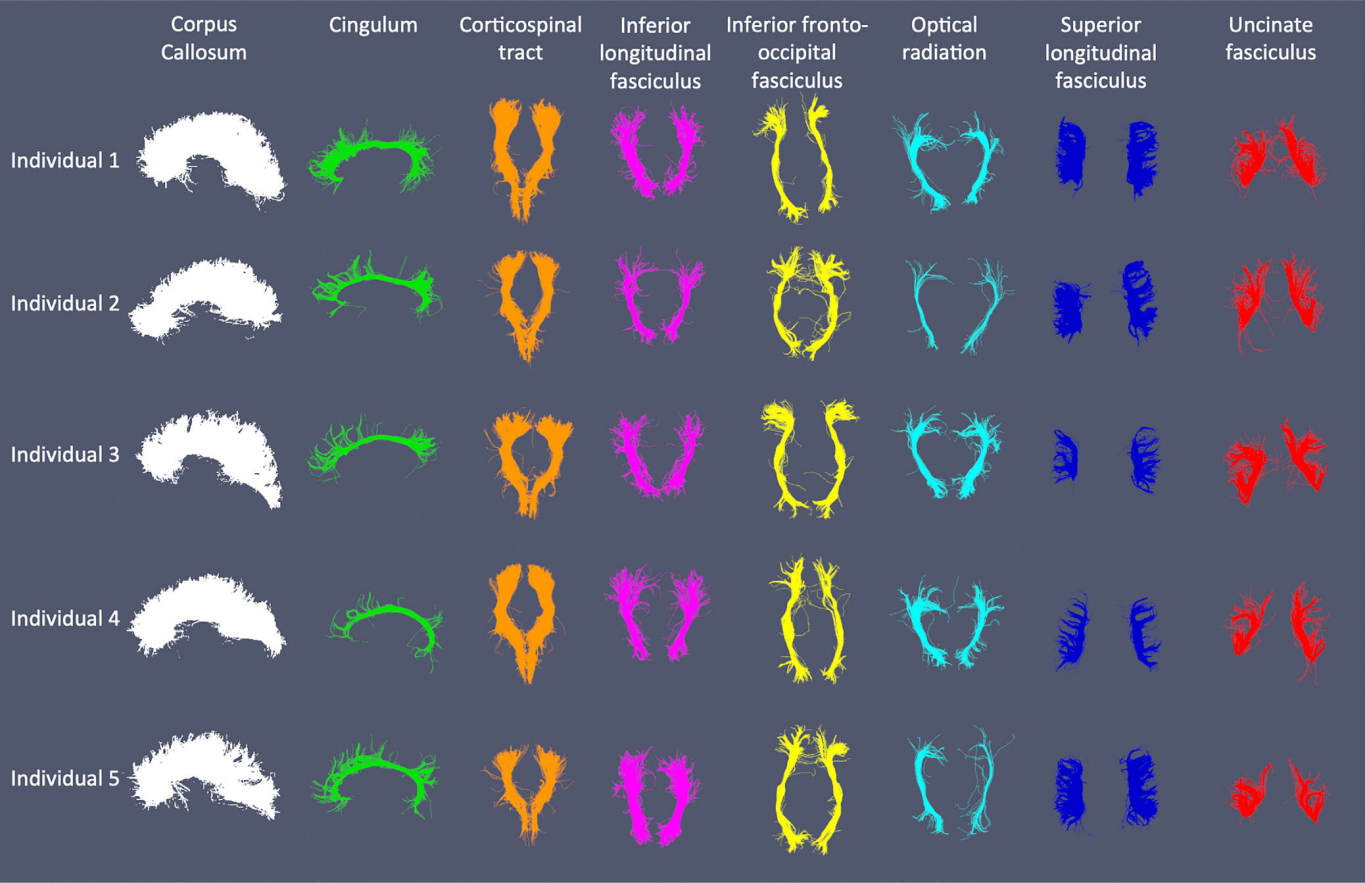
The eight white matter tracts resulting from the semiautomated tractography employed in the current study in a random selection of five of the 812 HUNT MRI individuals.

The semiautomated tractography of the eight major WM tracts (CC, CG, SLF, ILF, IFO, CST, OR, and UNC) in five of the 812 HUNT MRI participants is shown in Figure 4.

Similar to the TBSS analysis, the tractography ROI analyses demonstrated a positive association, with small effect sizes, between FA and ICV in all WM tracts except for OR (corrected for age, sex, and multiple comparisons) (Table 1). For OR, only a negative association was present in the right hemisphere (Table 1). A positive association was found between WM tract volume and ICV for all WM tracts, with slightly higher regression coefficients and medium effect sizes, compared to the FA versus ICV relationships (Table 1) (corrected for age, sex, and multiple comparisons).

**TABLE 1:** Relationships between ICV and mean FA and between ICV and white matter tract volumes from the semiautomated tractography for women and men jointly

White matter tract	FA			Volume		
	Regression coefficient	*p*‐Value	*η* ^2^	Regression coefficient	*p*‐Value	*η* ^2^
Corpus callosum	1.103E‐08	**.001**	0.014	0.078	**.000**	0.171
Cingulum	1.795E‐08	**.000**	0.029	0.022	**.000**	0.163
Corticospinal tract left	1.743E‐08	**.000**	0.027	0.026	**.000**	0.242
Corticospinal tract right	1.198E‐08	**.001**	0.012	0.025	**.000**	0.241
Inferior fronto‐occipital fasciculus left	7.345E‐09	**.034**	0.006	0.020	**.000**	0.158
Inferior fronto‐occipital fasciculus right	7.005E‐09	**.031**	0.006	0.019	**.000**	0.138
Inferior longitudinal fasciculus left	1.249E‐08	**.003**	0.011	0.017	**.000**	0.107
Inferior longitudinal fasciculus right	1.464E‐08	**.000**	0.019	0.015	**.000**	0.099
Optical radiation left	3.366E‐09	.510	0.001	0.006	**.000**	0.061
Optical radiation right	−2.115E‐08	**.000**	0.022	0.009	**.000**	0.104
Superior longitudinal fasciculus left	8.637E‐09	**.008**	0.009	0.023	**.000**	0.158
Superior longitudinal fasciculus right	1.544E‐08	**.000**	0.028	0.024	**.000**	0.159
Uncinate fasciculus left	1.363E‐08	**.000**	0.019	0.008	**.000**	0.061
Uncinate fasciculus right	7.194E‐09	**.039**	0.005	0.008	**.000**	0.081

*Note*: The linear regression models were corrected for age and sex while multiple comparisons were controlled using the Benjamini–Hochberg method. A corrected *p*‐value of <.05 was considered statistically significant and is shown in bold. *η* ^2^: Partial eta squared (effect size).

Abbreviations: FA, fractional anisotropy; ICV, intracranial volume.

### 3.3 DTI indices and sex

#### 3.3.1 Tract‐based spatial statistics

TBSS analysis of sex effects on DTI indices demonstrated both areas with significantly higher (*d* = 0.19) and lower FA (*d* = 0.20) in women compared to men when correcting for age (Figure 6a). FA was higher in women mainly in the association tracts in the occipital and parietal lobe (IFO, ILF, SLF), genu, and splenium of CC and fornix. The higher FA was due to lower RD (*d* = 0.21) (in 44% of voxels with higher FA in women) and higher AD (*d* = 0.19) (in 36% of voxels with higher FA in women) (results not shown). Lower MD (*d* = 0.24) was found in 15% of the voxels where FA was higher in women (results not shown). FA was higher in men mainly in the deep central WM structures and in association tracts in the frontal and temporal lobes (middle part of IFO, ILF, and SLF, UNC), external and internal capsule, frontal part of CG, deep parts of CST, which were mainly caused by lower RD (*d* = 0.21) (52% of voxels with higher FA in men) (Figure 6a), while 34% of the voxels with higher FA in men showed lower AD (*d* = 0.29) (results not shown). Lower MD (*d* = 0.19) was found in 38% of the voxels with higher FA in men (results not shown).

When correcting for both age and ICV, higher FA was only present in women compared to men, and the differences were even more widespread with 2.3 times more voxels significantly different (*d* = 0.13). The additional areas with higher FA in women compared to men were located in the central part of the WM association tracts (ILF, IFO, SLF, and UNC), body of CC and external and internal capsule (Figure 6b). The higher FA in women were accompanied with lower RD and higher AD in women when correcting for both age and ICV.

In the TBSS analysis comparing the ICV‐matched groups of women and men, differences in FA between the sexes were similar as in the analysis correcting for ICV, with 1.8 times more suprathreshold voxels in the same regions significantly higher in women compared to men in the age corrected analysis in the full sample (*d* = 0.12) (Figure 6c). There were no differences in MD, AD, and RD between women and men in the ICV‐matched subgroup analysis (results not shown). No areas with increased FA were found in men compared to women in the ICV‐matched subgroup analysis.

#### 3.3.2 Tractography

The semiautomated tractography demonstrated that women had higher FA than men in the CC, bilaterally in the IFO, ILF, SLF, and in the left OR and UNC (Table 2a), while there were no tracts with higher FA in men (corrected for ICV, age, and multiple comparisons). The opposite results were found for the WM tract volumes. They were significantly higher in men in CG, right CST, left ILF and UNC bilaterally (Table 2a), while no WM tracts had higher volumes in women compared to men (corrected for ICV, age, and multiple comparisons). The effect sizes were small for the sex difference in both tract FA and volume.

**TABLE 2:** Sex differences in mean FA (±SD) and white matter tract volumes (10^4^ × mm^3^) (±SD) between men (*n* = 383) and women (*n* = 429) from the semiautomated tractography analysis in the whole group (a) (corrected for age, ICV, and multiple comparisons) and in the ICV‐matched women and men (b) (corrected for age and multiple comparisons)

(a) Whole group								
White matter tract	FA				Volume			
	Women	Men	*p*‐Value	*η* ^2^	Women	Men	*p*‐Value	*η* ^2^
Corpus callosum	0.361 (0.011)	0.359 (0.011)	**.000**	0.022	12.46 (2.07)	13.91 (2.63)	.645	0.000
Cingulum	0.323 (0.012)	0.325 (0.013)	.216	0.002	1.93 (0.58)	2.59 (0.69)	**.000**	0.020
Corticospinal tract left	0.391 (0.013)	0.392 (0.012)	.120	0.003	3.31 (0.60)	3.75 (0.65)	.032	0.006
Corticospinal tract right	0.391 (0.013)	0.392 (0.013)	.576	0.000	3.13 (0.57)	3.48 (0.63)	**.000**	0.016
Inferior fronto‐occipital fasciculus left	0.347 (0.012)	0.342 (0.011)	**.000**	0.032	2.60 (0.54)	2.98 (0.67)	.804	0.000
Inferior fronto‐occipital fasciculus right	0.352 (0.011)	0.347 (0.011)	**.000**	0.041	2.45 (0.54)	2.84 (0.67)	.766	0.000
Inferior longitudinal fasciculus left	0.357 (0.015)	0.354 (0.014)	**.000**	0.020	2.15 (0.54)	2.61 (0.68)	**.017**	0.007
Inferior longitudinal fasciculus right	0.355 (0.013)	0.352 (0.012)	**.000**	0.031	2.18 (0.52)	2.54 (0.65)	.260	0.002
Optical radiation left	0.386 (0.016)	0.380 (0.017)	**.001**	0.015	0.97 (0.29)	1.08 (0.33)	.852	0.000
Optical radiation right	0.378 (0.017)	0.370 (0.016)	.149	0.003	0.87 (0.31)	1.01 (0.33)	.288	0.001
Superior longitudinal fasciculus left	0.333 (0.011)	0.328 (0.011)	**.000**	0.040	2.36 (0.61)	2.68 (0.75)	.698	0.000
Superior longitudinal fasciculus right	0.341 (0.011)	0.338 (0.011)	**.000**	0.047	2.41 (0.66)	3.01 (0.79)	.153	0.003
Uncinate fasciculus left	0.308 (0.012)	0.307 (0.011)	**.001**	0.014	0.95 (0.32)	1.28 (0.44)	**.000**	0.030
Uncinate fasciculus right	0.305 (0.012)	0.304 (0.011)	.032	0.006	0.94 (0.29)	1.26 (0.40)	**.000**	0.025

(b) ICV matched group								
White matter tract	FA				Volume			
	Women	Men	*p*‐Value	*η* ^2^	Women	Men	*p*‐Value	*η* ^2^
Corpus callosum	0.363 (0.011)	0.358 (0.011)	**.002**	0.036	13.06 (2.33)	12.86 (2.45)	.966	0.000
Cingulum	0.326 (0.012)	0.325 (0.013)	.482	0.002	2.22 (0.60)	2.34 (0.60)	.092	0.010
Corticospinal tract left	0.393 (0.012)	0.391 (0.011)	.157	0.007	3.61 (0.63)	3.51 (0.61)	.171	0.007
Corticospinal tract right	0.393 (0.012)	0.391 (0.012)	.387	0.003	3.42 (0.58)	3.23 (0.57)	**.004**	0.031
Inferior fronto‐occipital fasciculus left	0.348 (0.013)	0.341 (0.011)	**.000**	0.060	2.76 (0.55)	2.73 (0.66)	.983	0.000
Inferior fronto‐occipital fasciculus right	0.353 (0.011)	0.345 (0.011)	**.000**	0.108	2.60 (0.55)	2.59 (0.64)	.692	0.001
Inferior longitudinal fasciculus left	0.358 (0.014)	0.353 (0.015)	**.016**	0.021	2.29 (0.55)	2.37 (0.68)	.080	0.011
Inferior longitudinal fasciculus right	0.358 (0.013)	0.351 (0.012)	**.000**	0.062	2.29 (0.55)	2.35 (0.63)	.179	0.007
Optical radiation left	0.386 (0.018)	0.381 (0.016)	**.038**	0.016	1.01 (0.30)	0.97 (0.28)	.621	0.001
Optical radiation right	0.376 (0.017)	0.372 (0.017)	.166	0.007	0.97 (0.34)	0.90 (0.31)	.264	0.005
Superior longitudinal fasciculus left	0.334 (0.011)	0.328 (0.012)	**.000**	0.049	2.61 (0.61)	2.58 (0.64)	.695	0.001
Superior longitudinal fasciculus right	0.342 (0.011)	0.336 (0.011)	**.000**	0.069	2.68 (0.67)	2.70 (0.70)	.762	0.000
Uncinate fasciculus left	0.310 (0.011)	0.305 (0.011)	**.000**	0.047	1.04 (0.33)	1.20 (0.39)	**.000**	0.061
Uncinate fasciculus right	0.308 (0.012)	0.303 (0.010)	**.002**	0.033	1.03 (0.29)	1.14 (0.33)	**.001**	0.037

*Note*: Linear regression models, corrected for multiple comparisons using the Benjamini–Hochberg method. A corrected *p*‐value of <.05 were considered statistically significant and are shown in bold. *η* ^2^: Partial eta squared (effect size).

Abbreviations: FA, fractional anisotropy; ICV, intracranial volume.

The analyses of sex differences in WM tracts in the ICV‐matched group showed similar results as for the whole group, with higher FA in women, but with the addition of right UNC also having significantly higher FA in women compared to men (Table 2b) (corrected for age and multiple comparisons). The WM tract volume analyses demonstrated higher tract volumes in men only in bilateral UNC, while the volume of right CST was higher in women than men (Table 2b) (corrected for age and multiple comparisons).

### 3.4 DTI indices and age

#### 3.4.1 Tract‐based spatial statistics

A negative association was found between FA and age in all major WM tracts in women and men when correcting for sex (*β* = −1.5 × 10^−3^) (in 74% of all voxels in skeleton) (Figures 7 and 8). MD (*d* = 0.01) and RD (*d* = 0.02) increased with increasing age in almost the same areas as FA decreased (in 91 and 97% of voxels with decreased FA, respectively) (results not shown). AD increased with increasing age in 61% of the areas with decreased FA (*d* = 0.01) (results not shown). When correcting for both sex and ICV, highly similar associations between FA/MD/AD/RD, and age were found in similar areas (results not shown).

#### 3.4.2 Tractography

A significant negative association between FA and age was found in all WM tracts except for the left CST (Table 3a and Figure 8) (corrected for sex, ICV, and multiple comparisons). A significant negative association was also present between WM tract volume and age in all the WM tracts except for the bilateral CST and left SLF (Table 3a) (corrected for sex, ICV, and multiple comparisons). In general, the volume effects were larger than the FA effects, with medium effect sizes for the volume associations, and small effect sizes for the FA associations.

**TABLE 3:** Effect of age on WM tracts. Relationships between age and mean FA and between age and white matter tract volume from the semiautomated tractography for women and men jointly (corrected for sex and ICV)

	FA			Volume		
White matter tract	Regression coefficient	*p*‐Value	*η* ^2^	Regression coefficient	Volume *p*‐value	*η* ^2^
Corpus callosum	−0.00030	**.001**	0.013	−1702.62	**.000**	0.110
Cingulum	−0.00035	**.001**	0.014	−166.77	**.001**	0.015
Corticospinal tract left	−0.00006	.573	0.000	26.44	.563	0.000
Corticospinal tract right	−0.00022	**.039**	0.005	70.77	.108	0.003
Inferior fronto‐occipital fasciculus left	−0.00037	**.000**	0.018	−301.79	**.000**	0.052
Inferior fronto‐occipital fasciculus right	−0.00036	**.000**	0.019	−278.93	**.000**	0.043
Inferior longitudinal fasciculus left	−0.00050	**.000**	0.022	−257.00	**.000**	0.035
Inferior longitudinal fasciculus right	−0.00033	**.002**	0.012	−310.64	**.000**	0.055
Optical radiation left	−0.00038	**.007**	0.009	−141.27	**.000**	0.040
Optical radiation right	−0.00049	**.001**	0.015	−130.35	**.000**	0.033
Superior longitudinal fasciculus left	−0.00051	**.000**	0.038	−100.50	.056	0.005
Superior longitudinal fasciculus right	−0.00041	**.000**	0.024	−132.72	**.018**	0.007
Uncinate fasciculus left	−0.00039	**.000**	0.020	−160.88	**.000**	0.033
Uncinate fasciculus right	−0.00068	**.000**	0.057	−82.59	**.003**	0.011

*Note*: Linear regression models, corrected for multiple comparisons using the Benjamini–Hochberg method. A corrected *p*‐value of <.05 were considered statistically significant and are shown in bold. *η* ^2^: Partial eta squared (effect size).

Abbreviations: FA, fractional anisotropy; ICV, intracranial volume; WM, white matter.

## 4 DISCUSSION

This is one of the largest general population‐based studies performed on the same scanner with the same software to investigate the role of ICV and sex as well as aging on four commonly used DTI indices (FA, MD, RD, and AD) using a healthy cohort of middle‐aged adults between 50 and 66 years of age. ICV was similarly associated with the DTI indices in men and women. Sex differences were uncovered in the ICV‐matched samples, demonstrating higher FA and lower MD in women mainly in posterior parts of the intrahemispheric tracts, as well as lower uncinate tract volumes. Combined these findings demonstrated that both ICV and sex can contribute to variation in WM microstructure as measured by DTI. Even the narrow age‐range in this study (50–66 years) was associated with lower FA across the entire WM accompanied by higher MD and RD values, likely reflecting loss of axonal integrity, myelination, cellular packing or a combination of these. There was neither a clear anterior–posterior/superior–inferior gradient nor circumscribed changes located to particular tracts for FA in this age group in the last decade before old age. The results from the voxel based and the tractography/ROI methods were similar, both with small effect sizes, indicating similar sensitivity. The results also suggest that the associations between DTI indices and ICV, age and sex are equally important for voxel‐based and tractography/ROI methods.

### 4.1 ICV and WM

ICV had a marked and similar association with DTI indices in men and women. TBSS and tractography results demonstrated that individuals with larger ICV have higher FA, regardless of sex and age. To our knowledge, only two previous studies have explicitly investigated and demonstrated a similar positive association between FA and ICV in a large group of adults between 24 and 84 (Takao et al., 2011) and 40 and 49 (Takao et al., 2014) years of age. Both studies showed a lower number of areas with significant FA‐ICV associations compared to our study, with the Takao et al.'s study from 2014 showing the lowest number of associations. This could be explained by the fact that both studies used a significance threshold of *p* < .025, which was lower than used in our study. Furthermore, the study from 2014 included 238 individuals, which is considerably lower than the 812 and 821 included in our study and in the study of Takao et al. (2011), respectively. The link between ICV and FA is also consistent with a recent study showing a positive association between total brain volume and FA in three independent data sets of 15‐, 19‐, and 28‐year‐olds acquired with a resolution of 1.25–2 mm (Warling et al., 2021). Taken together, a small, but significant association between ICV and DTI indices can be observed over a wide range of age and image resolutions suggesting that ICV should be considered as a potential variable to be included in the statistical model.

The increase in FA associated with larger ICV was mainly accompanied by lower RD and to a lesser extent lower MD. These associations suggest that a larger ICV could be linked to more axons, better myelination, and greater axonal packing based on previous histological animal and human studies (Seehaus et al., 2015; Schmierer et al., 2007; Song et al., 2005; Lazari & Lipp, 2021). The theoretical framework used in studies of brain size on brain microstructural anatomy derives from studies of evolution which show that WM volume is disproportionally enlarged with larger brain size, partly due to presence of more well‐myelinated axons and some axons with very large diameter (Bush & Allman, 2003; Kaas, 2000). It is a stretch to use this framework to explain how variation in ICV within a species is linked to differences in (micro)anatomy. As far as we know, no histological studies have compared WM microstructure within a species (human or animal) based on ICV or brain size. The consistent findings of higher FA and lower RD and MD in this study and the three previous studies examining associations between DTI indices and ICV in different age groups and at different resolutions (Takao et al., 2011; Takao et al., 2014; Warling et al., 2021), suggest that differences in ICV is accompanied by consistent differences in DTI indices considered to reflect WM microstructure. Although speculative, a larger brain might require larger axonal diameter and higher degree of myelination to maintain conduction time along the longer axons (Liewald et al., 2014), and more intrahemispheric connections as long‐range connections become increasingly inefficient in larger brains (Ringo et al., 1994). Support for this theory has been found in humans, where larger brains have greater intrahemispheric connectivity, as measured with DTI, than smaller brains, independent on sex (Hänggi et al., 2014). Finally, the spatially heterogeneous expansion of WM (Warling et al., 2021) and cortical surface area (Reardon et al., 2018) with increasing brain size may also lead to subtle changes in WM microstructure that may be observed using DTI. Our tractography results support this by showing that in addition to the ICV‐FA effect; ICV was associated with tract volume. The relationship between ICV and tract volume was more notable than for FA, in agreement with a recent study showing that regional WM volumes increase more strongly with total brain volume than FA (Warling et al., 2021).

The associations between ICV and DTI indices may also be linked to partial volume effects. The voxel of a small brain could include more complex fiber configurations and more tissue types compared to a similar voxel in a large brain, leading to a lower FA in those with smaller ICV. Likewise, smaller partial volume effects are expected for the larger brains, which could lead to increased FA and decreased MD in the same voxel. A positive association between FA and spatial resolution has been demonstrated in several studies, supporting a role of partial volume effects for the association between ICV and FA (Fujiwara et al., 2008; Kim et al., 2006; Oouchi et al., 2007; Papanikolaou et al., 2006). Since the same positive association between ICV (total brain size) and a higher FA was found in our study and a study including three large neuroimaging data sets with resolutions varying between 1.25 and 2.5 mm (Warling et al., 2021), partial volume effects and/or ICV effects seems to be pervasive. Even if the associations between ICV and the DTI indices are due to partial volume effects alone, it does not negate the relevance of including ICV as a variable in studies comparing men and women, and neurodevelopmental diseases and disorders such as attention deficit hyperactivity disorder (Hoogman et al., 2017), Fabry disease (Pontillo et al., 2018), autism spectrum disorders (Hiess et al., 2015), premature birth (Aanes et al., 2019), and neurogenerative disorders where sex differences in prevalence are well known (Dubal, 2020).

### 4.2 Sex and WM

**FIGURE 5:**
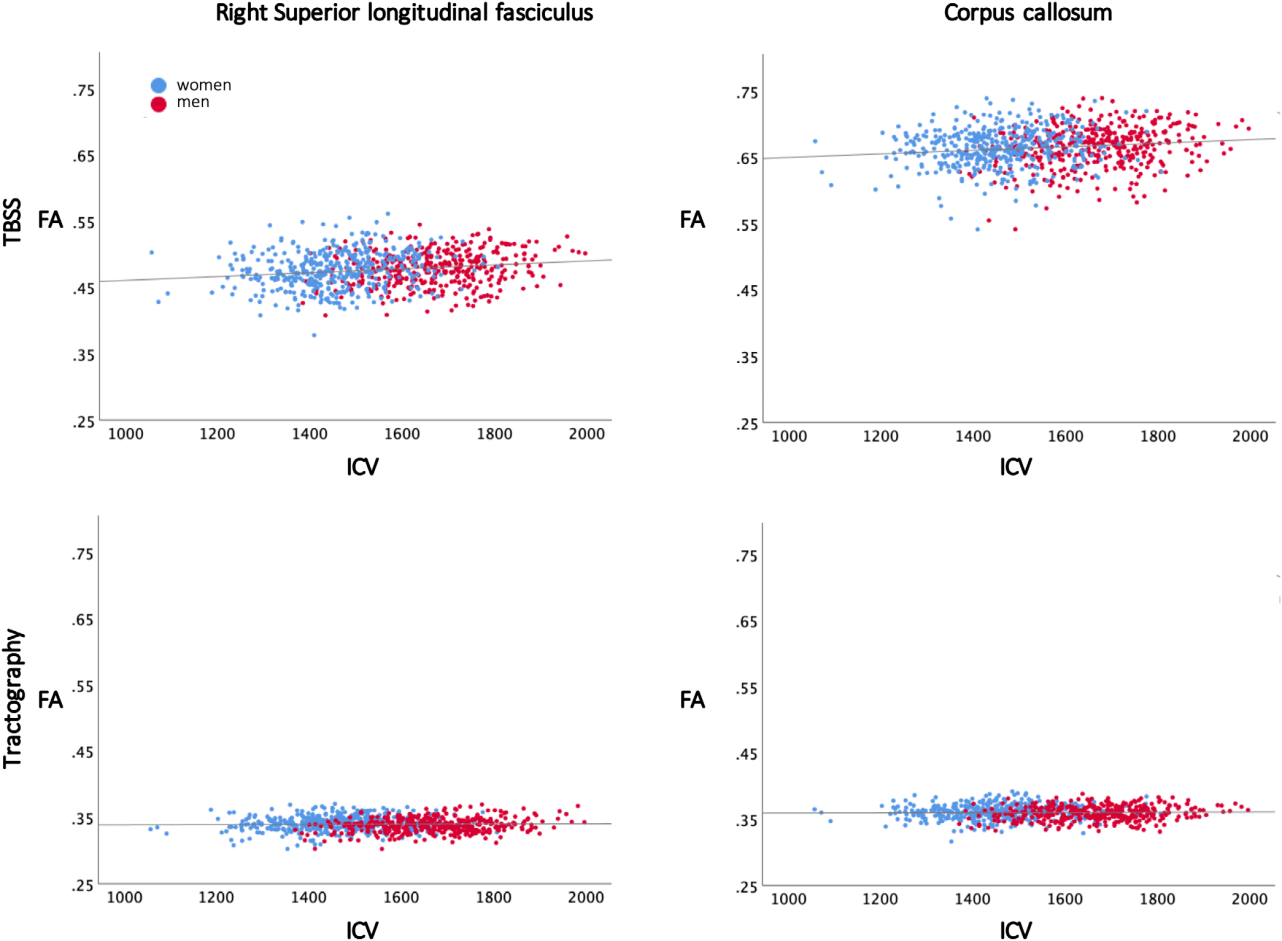
Intracranial volume (ICV) plotted against fractional anisotropy (FA) for two of the white matter tracts in the study, the right superior longitudinal fasciculus and corpus callosum, from the tract‐based spatial statistics (TBSS) (top row) and tractography regions of interest (ROIs) (bottom row). The women (blue) and men (red) are plotted in different colors, while the regression line is the same for both sexes since the slope did not differ. For the TBSS data, the anatomical ROIs were obtained from the JHU ICBM DTI 81 atlas in FSL. Only voxels with significant associations were used to compute the mean FA values. For the tractography ROI data, the mean value for each of the tractography ROIs for each individual were plotted.

The literature on sex differences in DTI indices is contradictory, which is easy to understand when looking at the results in Figure 5. Our study shows that there are associations between DTI‐based indices of WM microstructure and both ICV and sex, as detected in the ICV‐matched sample. This can explain some of the controversies in the literature. When controlling for age, sex differences in FA were found in large areas of the WM skeleton in the TBSS analysis. Most areas had higher FA in women compared to men, mainly in the association tracts in the occipital and parietal lobe and genu and splenium of CC. Substantial areas with higher FA in men compared to women were also found, mainly in the deep central WM structures and in association tracts in the frontal and temporal lobes. The areas with higher FA in men compared to women resembles the results from another large cross‐sectional study investigating sex differences in 857 healthy adults between 25 and 85 years (Inano et al., 2011). In the same study, women only had higher FA in the fornix compared to men (Inano et al., 2011), while large posterior–superior WM regions had higher FA in our study using the same type of analysis (TBSS) (Figure 5a). The large difference in results between these two studies with approximately equal number of individuals could be due to differences in the age span included (middle‐aged adults between 50 and 66 vs. 25 and 85 years).

**FIGURE 6:**
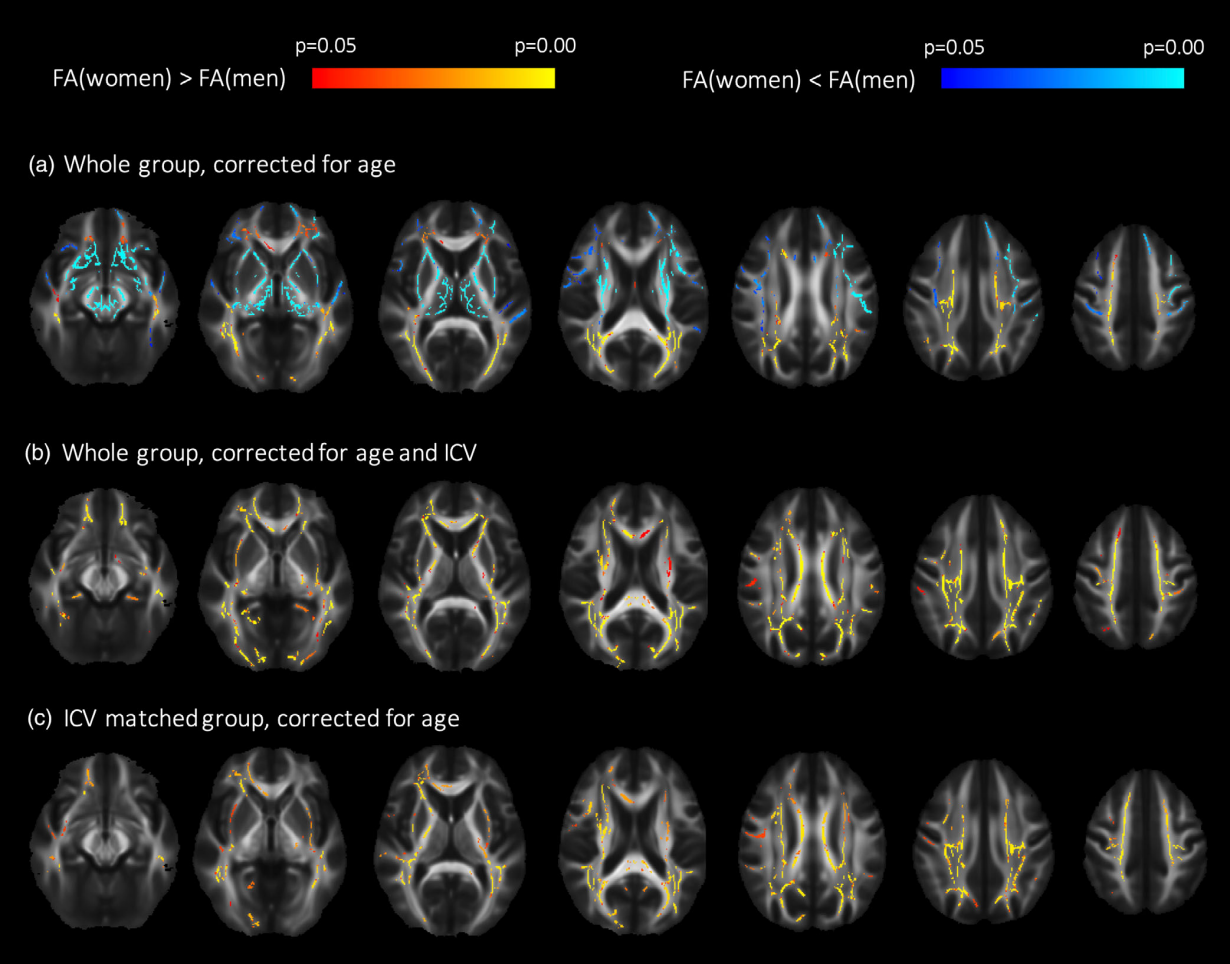
Sex differences in diffusion tensor imaging (DTI) indices. (a) Tract‐based spatial statistics (TBSS) analysis demonstrated both areas with significantly higher (red/yellow) and lower (blue/light blue) fractional anisotropy (FA) in women compared to men when correcting for age in the whole group. (b) When correcting for age and intracranial volume (ICV) in the whole group only women had areas with higher FA compared to men. (c) The group comparison between the ICV‐matched men and women (correcting for age) uncovered only areas with higher FA in women compared to men. A corrected *p*‐value of <.05 were considered significant.

**FIGURE 7:**
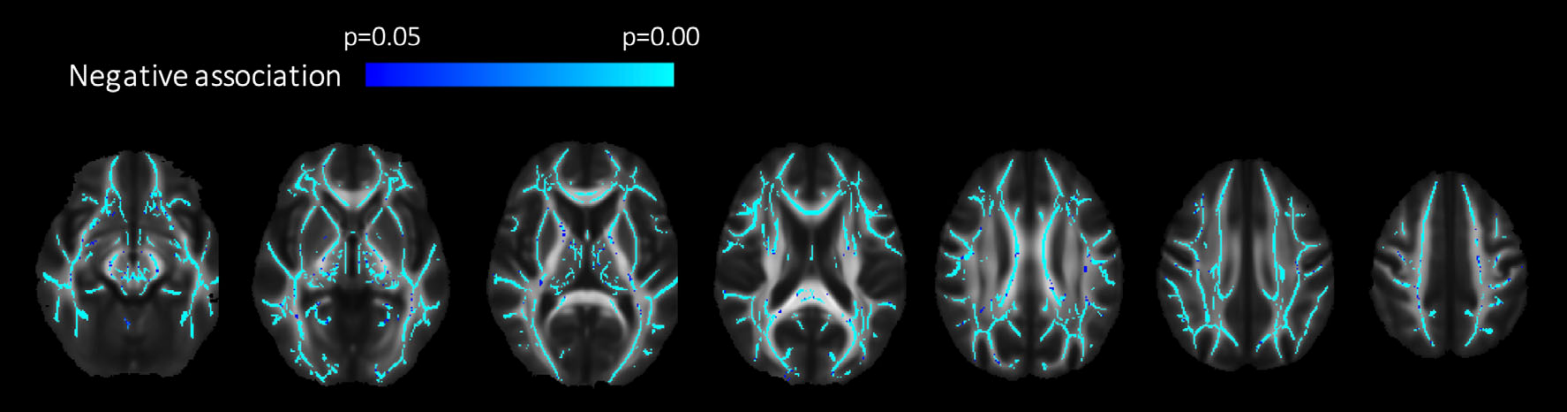
Effect of age on fractional anisotropy (FA). A negative association was found between FA and age in all major white matter tracts in women and men (*p* < .05, corrected for sex and multiple comparisons).

**FIGURE 8:**
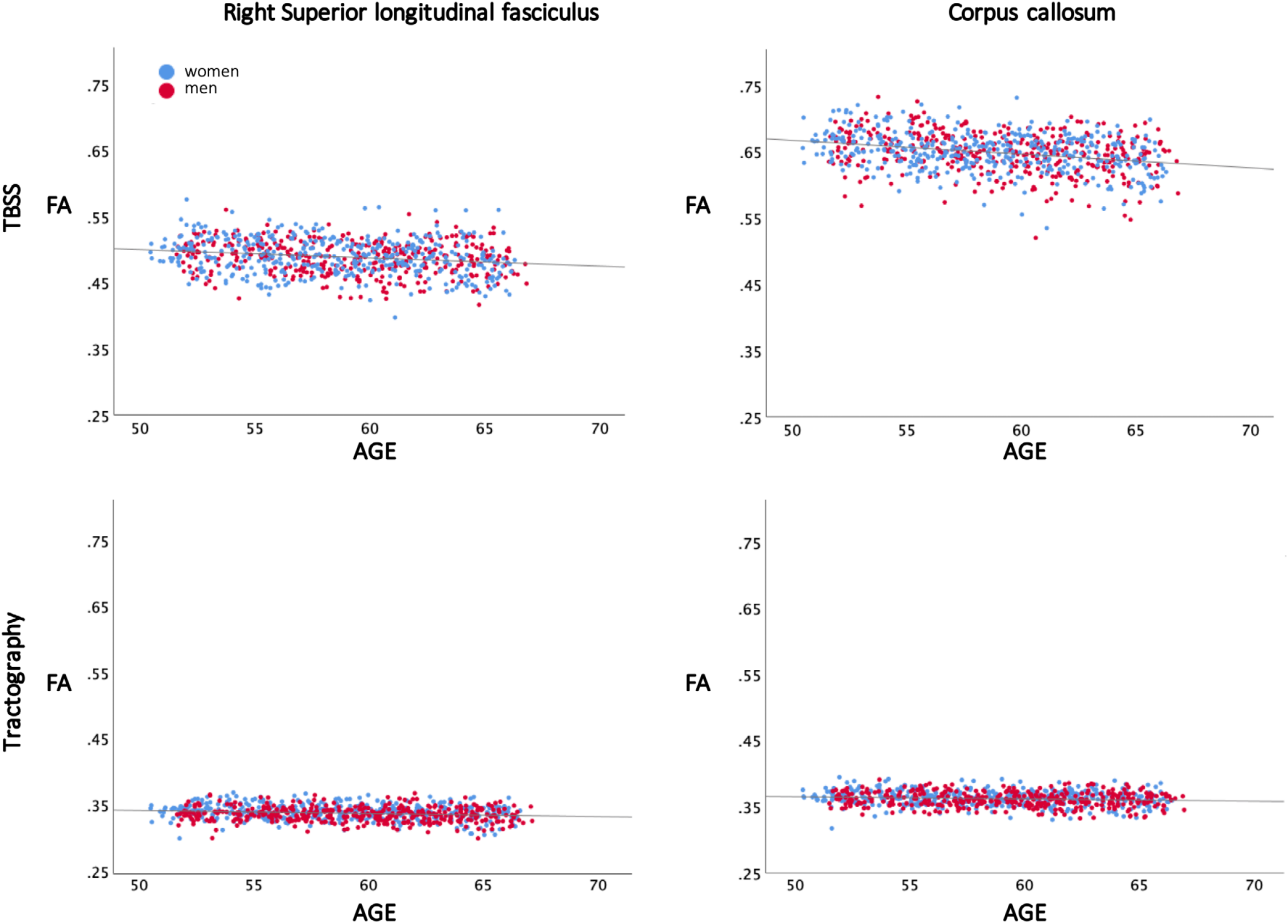
Age plotted against fractional anisotropy (FA) for two of the white matter tracts in the study, the right superior longitudinal fasciculus and corpus callosum, from the tract‐based spatial statistics (TBSS) (top row) and tractography regions of interest (ROIs) (bottom row). The women (blue) and men (red) are plotted in different colors, while the regression line is the same for both sexes since the slope did not differ. For the TBSS data, the anatomical ROIs were extracted from the TBSS skeleton using the JHU ICBM DTI 81atlas in FSL. Only voxels with significant associations were selected and used in the plots. For the tractography ROI data, the mean value for each of the tractography ROIs for each individual were plotted.

Other smaller studies report higher FA in women compared to men in the SCR and CST in adolescents (Bava et al., 2011), and in the splenium of CC in healthy individuals with a mean age of 35 years (Gongora et al., 2016) and 66 years (O'Dwyer et al., 2012). Studying specific tracts, Huster et al. (2009) and Lebel et al. (2010) demonstrated higher FA in men compared to women in CG, while Oh et al. (2007) found both higher FA in men and women in different parts of CC. Except for the CST results, all the above results are in line with the results from our study, but with the difference that we found substantially more areas with significant differences in both men versus women and women versus men. Furthermore, many studies do not detect sex differences in DTI parameters (Burzynska et al., 2010; Salami et al., 2012). In addition to differences in DTI acquisitions and methods for analyzing the DTI data, there could be several explanations for the discrepancies in the sex‐related differences in FA. First, the TBSS and tractography results from our study show that the differences and effect sizes are small, which indicate that large studies are needed to show sex‐related differences in the DTI parameters. Furthermore, none of the previous studies investigating sex differences have included individuals in the same narrow age range as the current study, and there is a substantial variety in the participant's age span in the previous studies. Although correcting for age, a wide age span could affect the results, as the sex differences in FA could change during development/early adulthood. FA increases through childhood and adolescence, reaching a plateau around age 30 years, followed by a decrease starting around age 40–50 years depending on brain region (Lebel et al., 2012). Studies including a large age span from the 20s to the 40s will therefore incorporate both an FA increase and decrease, making it difficult to draw conclusions regarding the sex differences at the varying ages and to compare with studies that have a narrower age span as in our study. Our study has the advantage of a narrow age range, which will minimize the contribution of developmental effects.

Interestingly, when correcting for ICV and when analyzing sex differences in the ICV‐matched groups in the TBSS analysis, no area with higher FA in men compared to women was found. Moreover, the areas with higher FA in women increased compared to the analysis in which ICV was not controlled for. Despite fewer subjects, the sex effect in FA in the ICV‐matched groups was spatially more distributed in the TBSS analysis and had larger effect sizes in the tractography analysis. These results show the presence of sex differences in FA independent of ICV. The higher FA value was accompanied by decreased RD and increased AD, suggesting that women have higher WM microstructural integrity than men with similar brain volumes. This may be caused by greater myelination and density of tracts, and a more coherent WM tract organization or a combination of these factors.

In line with our results, two other studies have observed an effect of sex on DTI indices when correcting for ICV. Takao et al. (2014) demonstrated a significant reduction of areas with sex‐related differences in FA in their TBSS analysis, while Choi et al. (2010) found that the sex related difference in MD in the temporal lobe disappeared in their tractography ROI analysis. When examining structural brain differences between men and women, it is crucial to correct for other factors that may influence these differences. In volumetric studies on sex differences, ICV is commonly used as a covariate to account for differences due to brain size as it is well‐known that there are on average, volumetric differences between the sexes (Pintzka et al., 2015). ICV is seldom used as a covariate in DTI studies, presumably because it is assumed that DTI indices are not associated with ICV. Of the previous studies on sex differences in DTI mentioned above, only three controlled for ICV (Choi et al., 2010; Pietrasik et al., 2020; Takao et al., 2014). This could mean that the results from many studies on sex‐related WM differences could in part be explained by differences in brain size, and not sex per se. Our results demonstrate the importance of including ICV, or a similar measure of brain size, when studying WM differences using DTI, to ensure that we are not studying larger versus smaller brains, but the actual question of interest.

In contrast to the extensive sex‐related FA differences in the TBSS analysis in the whole sample found in the current study, higher tract volumes were only found in men in a few WM tracts (CG, right CST, left ILF, and UNC bilaterally). In the ICV‐matched group, volume differences were only found in two WM tracts, with higher volumes in women in the right CST and higher tract volumes in men bilaterally in UNC. The observation that sex differences in FA did not overlap with the tract volume differences makes it plausible that microstructural rather than macroscopic differences in WM cause the sex‐related FA differences. The tract volume result corresponds to studies investigating sex‐related differences in subcortical volumes. When correcting for ICV, the sex‐related subcortical volume differences were reduced (Pintzka et al., 2015) or disappeared (Jancke et al., 2015), indicating that subcortical and WM tract volume differences are mainly affected by brain size and not biological sex.

### 4.3 Age and WM

Even within the limited age range of 16 years, widespread negative associations were uncovered for the DTI indices suggesting reduced WM microstructural integrity. It should be noted though that the effect sizes were small. Nevertheless, age affected more voxels in the WM skeleton than ICV, but age and ICV had comparable effect sizes according to the eta squared calculations from the tractography ROI analysis. A lower FA with increasing age was present in all major WM tracts in both the TBSS and tractography analyses. Controlling for ICV did not change the relationship between FA and age. There was also no interaction between ICV and age on the DTI parameters. Lower FA with age is in line with most other studies of the aging brain as shown in cross‐sectional (Cox et al., 2016; de Groot et al., 2015; Lawrence et al., 2020; Lebel et al., 2012; Salami et al., 2012; Sexton et al., 2014) and longitudinal studies (de Groot et al., 2016; Sullivan et al., 2010). The age‐related reduction in FA coincides with postmortem histological studies showing that healthy aging is accompanied by axonal and myelin degeneration (Aboitiz et al., 1996; Marner et al., 2003; Meier‐Ruge et al., 1992; Peters, 2002; Tang et al., 1997). The negative association between FA and age in our study was accompanied by an increase in RD in almost all voxels with lower FA in the TBSS analysis, suggesting that demyelination may be the main explanation for the age‐related changes in the DTI indices. Animal studies suggest that a decrease in AD correlates with axonal degeneration and less coherent tract organization (Song et al., 2003). However, the FA decline in our study was accompanied by an increase in AD in 61% of voxels with FA decline, which is in line with previous studies reporting two patterns of age‐related diffusion changes; FA decreases with increase in RD only or FA decrease with increase in both RD and AD (Inano et al., 2011; Vernooij et al., 2008). Increased longitudinal diffusion (i.e., higher AD) with age may be due to greater extracellular water, myelin‐ or axonal loss or a combination of these factors (Burzynska et al., 2010; Sexton et al., 2014). The present age‐related FA reduction, together with previous studies, demonstrates that this DTI index is a particular sensitive marker of age‐related WM changes.

Although most studies are consistent regarding the age‐related FA decline, there are differences in the spatial distribution of the WM changes and the onset of the decline. One explanation for these diverging findings could be differences in the age range in different studies. Most studies have included a wide age span (24–85, 5–59, 44–77, 30–80, 18–85, 65–91, and 45–100 years) incorporating both the FA increase connected to development and FA decrease connected to both late development and aging, compared to our study (50–66 years), which will only include an FA decrease (Lebel et al., 2012). Further, it has been shown that the onset and magnitude of changes in DTI indices in the aging brain differ depending on location in the brain and on the age‐interval under investigation (Lebel et al., 2012). Cross‐sectional studies with a wide age range also show a gradually steeper decrease in FA with increasing age (de Groot et al., 2016; Hsu et al., 2010; Sexton et al., 2014). Compared to many other studies, our participants are at the onset of the steeper WM decline expected to take place after age 65. Still our sample displayed extensive effects of age on FA through the whole WM, demonstrating that the FA decline affected all of WM by age 66.

Different theories exist regarding which WM areas are most sensitive to aging and how the affected areas change with age. These theories include anterior–posterior gradient (or last‐in‐first‐out hypothesis) (Bennett et al., 2010; Brickman et al., 2012; Burzynska et al., 2010; Cox et al., 2016; Michielse et al., 2010; Salat et al., 2005), a superior–inferior gradient (Sexton et al., 2014; Sullivan et al., 2010; Zahr et al., 2009), as well as a selective deterioration of specific WM tracts (Greenwood, 2000; Salat, 2011). Our results do not support any of these theories, as we found widespread FA decline in all parts of WM in the TBSS analysis and all the tracts in the ROI analysis except the CST.

No interaction effect was found between age and sex in the current study, consistent with one of the other large population‐based studies (Inano et al., 2011), while other similar DTI studies did not examine interactions (Burzynska et al., 2010; de Groot et al., 2016; Salami et al., 2012). A few studies have, on the other hand, shown an interaction between age and sex, demonstrating either a faster decline in WM integrity in men compared to women (Abe et al., 2010; Kochunov et al., 2012; Kumar et al., 2013) or a significantly greater linear relationship between age and FA in men compared to women (Sexton et al., 2014). However, the larger age‐span in these studies (20–80 years) makes it difficult to compare with our results.

A negative association was also present between WM tract volume and age in all the WM tracts except the bilateral CST and left SLF. The reduction in tract volume is probably caused by loss of axons and myelin, as discussed above. In the analysis in the ICV‐matched group, fewer tracts were associated with age, which could be caused by a smaller sample and a different selection of participants. Nevertheless, the negative association between WM tract volume and age is in line with previous studies demonstrating the same association (Salat et al., 2009).

### 4.4 Limitations and implications of the study

All the factors investigated in this large population‐based study were shown to be associated with DTI indices. Although the effects were significant, it is important to note that the associations and group differences were small showing that a large sample size is needed to uncover these associations and differences. Results from the voxel‐based and tractography analysis are largely in agreement, demonstrating the same associations between ICV, sex, and age and the DTI indices. Our results, therefore, demonstrated no added value of including both TBSS and tractography analysis of the DTI data in the current data set, except for adding the tract volume information and increasing the trustworthiness of the results. There are also a number of methodological limitations with the TBSS method, which are important to be aware of (Bach et al., 2014). Although visual quality control was done on the registered FA maps, subtle registration errors may still be present which may have influenced our findings in an unpredictable manner. The TBSS skeleton is not always the center of a single WM tract but may be a composite of several tracts. This aspect may be particularly relevant in relation to ICV, as the merging and diverging of separate tracts on the skeleton might to some degree depend on brain size. Finally, the TFCE multiple comparison correction method used for the voxel‐wise TBSS statistics, may be overly dependent on neighboring voxels so that small changes propagate over the skeleton rendering large parts either significant or nonsignificant (Bach et al., 2014).

## 5 CONCLUSION

A positive association was found between ICV and FA in all major WM tracts, equal in both men and women. Only women had regions with higher FA compared to men when correcting for ICV, and in the ICV‐matched group analysis, indicating that sex‐related differences are present in DTI indices suggesting presence of sex‐related differences in WM microstructure. Since both ICV and sex were shown to be related to DTI indices of WM, both factors should be considered in statistical analysis of DTI data. There are several conditions in which either men or women are more commonly affected, and/or where brain size is affected. How to control for ICV and sex in DTI analysis remains to be explored in future studies and will depend on for instance participant characteristics and research question. The age‐related FA decline was found to be similar for men and women, independent of ICV and affecting the entire WM similarly with no gradients or predilection for loss of WM microstructure.

## Supporting information


**FIGURE S1** A positive association (red/yellow) was found between FA and ICV in all major white matter tracts in women and men, while a negative association was found between ICV and MD and between ICV and RD (blue/light blue). For AD a mix of both positive (red/yellow) and negative (blue/light blue) associations was found. The color bars show voxelwise maps with the range of unstandardized beta values for the statistical significant voxels from the TBSS analysis (*p* < .05, corrected for age, sex and multiple comparisons).

**FIGURE S2** TBSS analysis demonstrated both areas with significantly higher (red/yellow) and lower (blue/light blue) FA in women compared to men when correcting for age and multiple comparisons in the whole group (a). When correcting for age, ICV and multiple comparisons in the whole group (b) and when performing the analysis in the ICV matched subgroup correcting for age and multiple comparison (c) FA was found to be higher only in women compared to men. The color bars show voxelwise maps with the range of Cohen's *d* for the statistical significant voxels from the TBSS analysis (*p* < .05, corrected for multiple comparisons).

**FIGURE S3** A negative association was found between FA and age in all major white matter tracts in women and men. The color bar show the voxelwise map with the range of unstandardized beta values for the statistical significant voxels from the TBSS analysis (*p* < .05, corrected for sex and multiple comparisons).

## Data Availability

The data sets generated and/or analyzed during the current study are not publicly available due to the European Union General Data Protection Regulations (GDPR), but are available from the corresponding author on reasonable request.
