# Supplementary figures and images for: Both brain size and biological sex contribute to variation in white matter microstructure in middle‐aged healthy adults
Source: Hum Brain Mapp. 2022 Oct 3;44(2):691–709. doi: 10.1002/hbm.26093 (PMC9842919; doi:10.1002/hbm.26093)

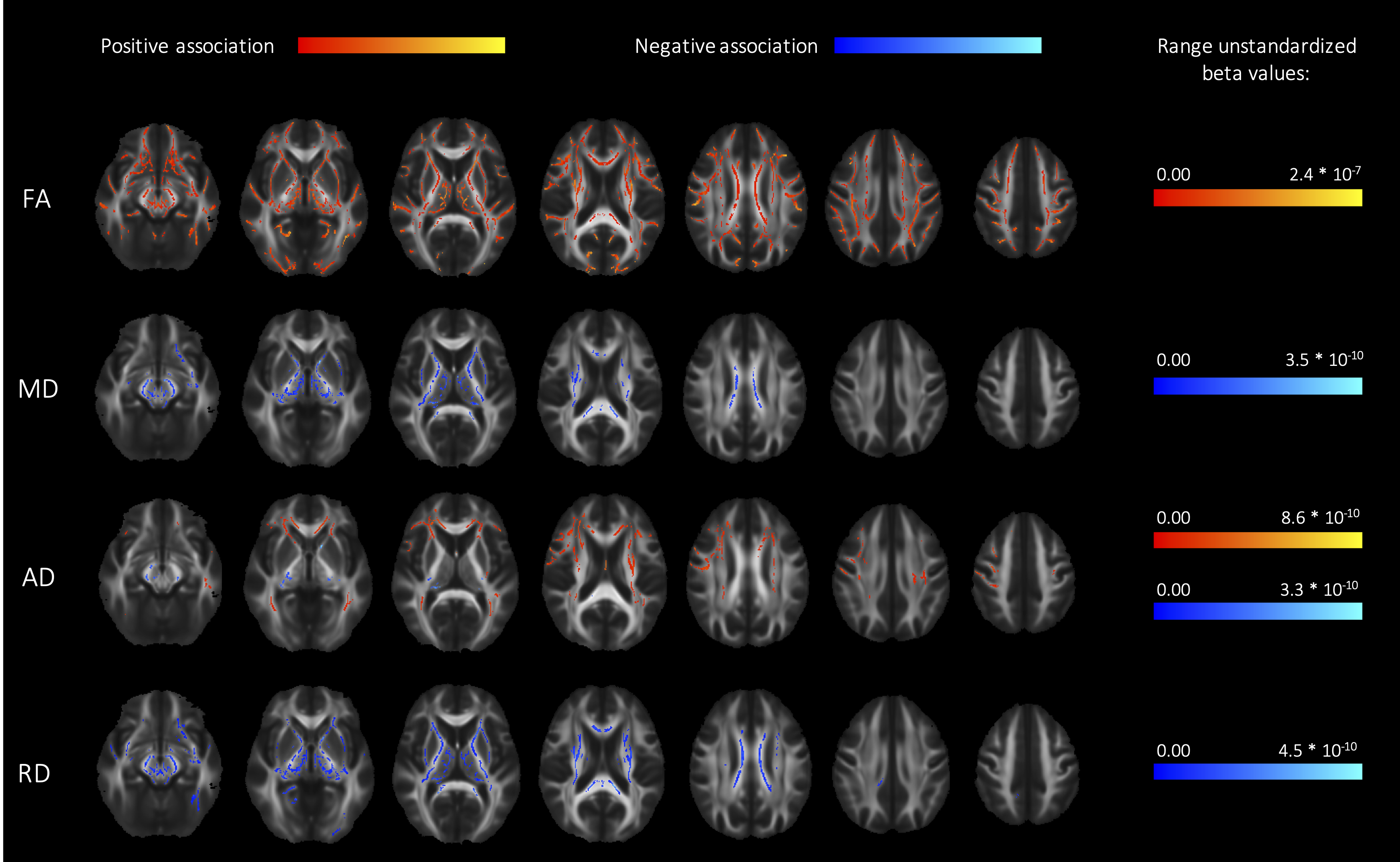

Supplement: Supplementary file 1 — FIGURE S1 A positive association (red/yellow) was found between FA and ICV in all major white matter tracts in women and men, while a negative association was found between ICV and MD and between ICV and RD (blue/light blue). For AD a mix of both positive (red/yellow) and negative (blue/light blue) associations was found. The color bars show voxelwise maps with the range of unstandardized beta values for the statistical significant voxels from the TBSS analysis (p < .05, corrected for age, sex and multiple comparisons). [file HBM-44-691-s001.tiff]

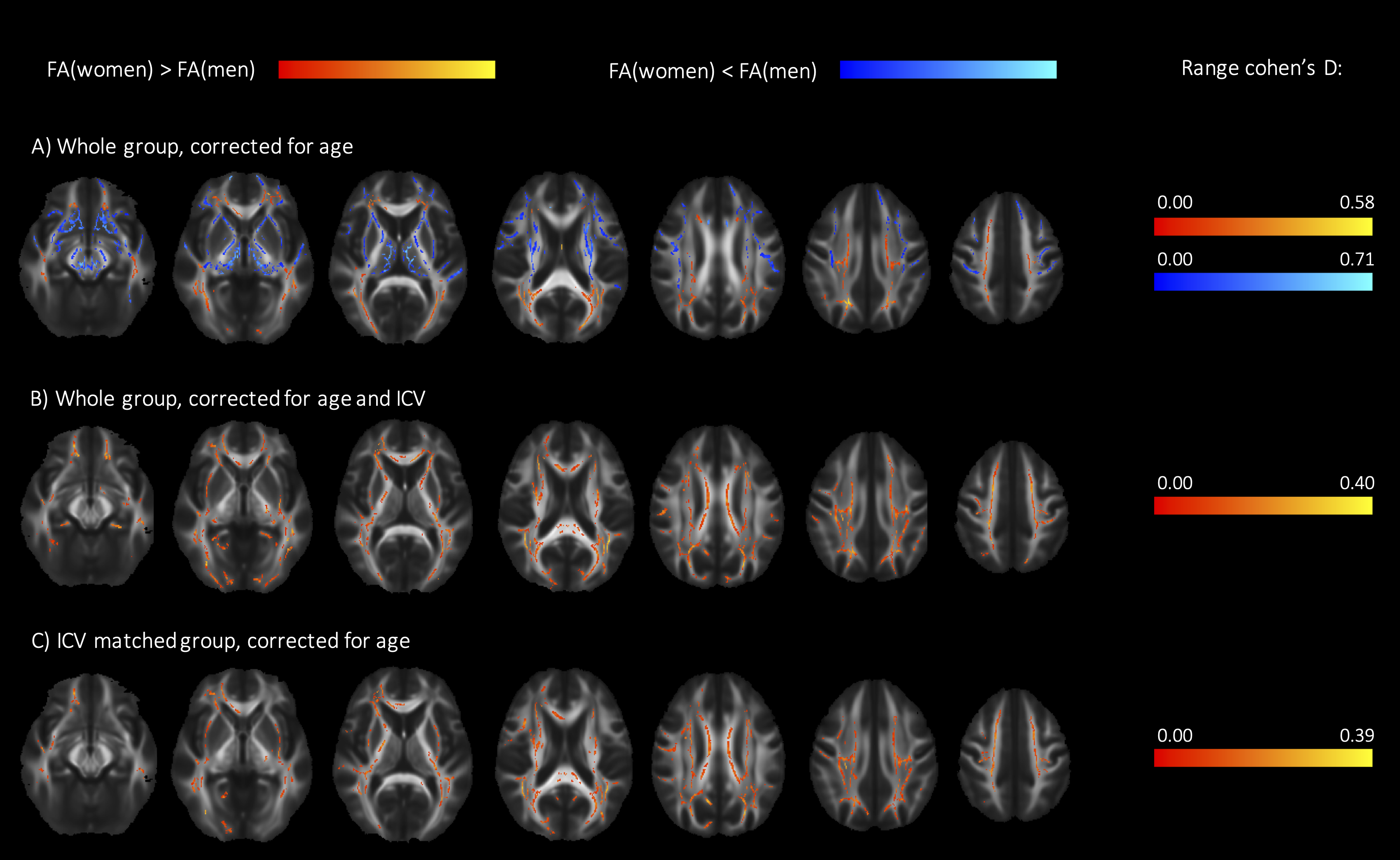

Supplement: Supplementary file 2 — FIGURE S2 TBSS analysis demonstrated both areas with significantly higher (red/yellow) and lower (blue/light blue) FA in women compared to men when correcting for age and multiple comparisons in the whole group (a). When correcting for age, ICV and multiple comparisons in the whole group (b) and when performing the analysis in the ICV matched subgroup correcting for age and multiple comparison (c) FA was found to be higher only in women compared to men. The color bars show voxelwise maps with the range of Cohen's d for the statistical significant voxels from the TBSS analysis (p < .05, corrected for multiple comparisons). [file HBM-44-691-s003.tiff]

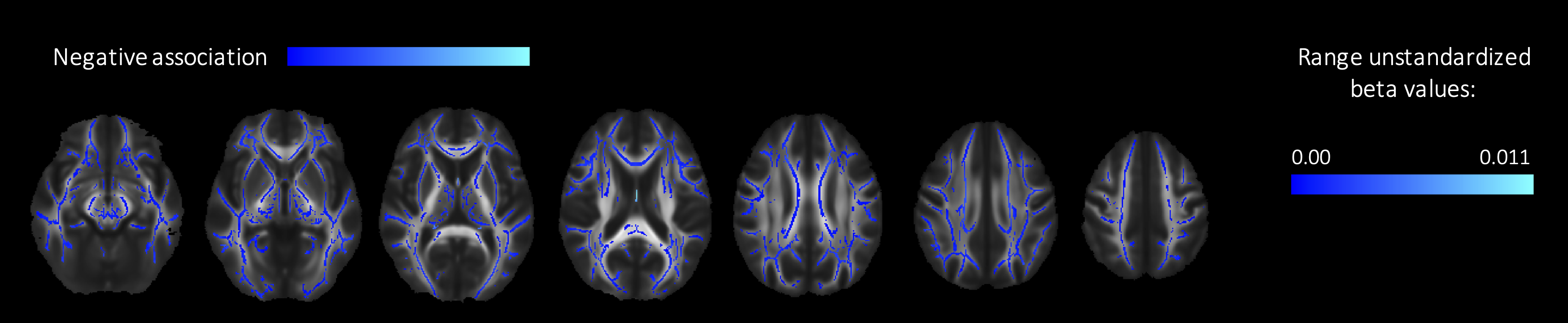

Supplement: Supplementary file 3 — FIGURE S3 A negative association was found between FA and age in all major white matter tracts in women and men. The color bar show the voxelwise map with the range of unstandardized beta values for the statistical significant voxels from the TBSS analysis (p < .05, corrected for sex and multiple comparisons). [file HBM-44-691-s002.tiff]
